# Uterine tumor resembling ovarian sex cord tumor (UTROSCT), case report with literature review

**DOI:** 10.1186/1755-7682-7-47

**Published:** 2014-11-04

**Authors:** Atif Ali Hashmi, Naveen Faridi, Muhammad Muzzammil Edhi, Mehmood Khan

**Affiliations:** Department of Histopathology, Liaquat National Hospital and Medical College, Karachi, Pakistan; Liaquat National Hospital and Medical College, Karachi, Pakistan; Dhaka Medical College, Dhaka, Bangladesh

**Keywords:** Uterine tumors resembling ovarian sex cord tumors (UTROSCT), Endometrial stromal tumor with sex cord like elements (ESTSCLE), CD99

## Abstract

**Introduction:**

Uterine tumors resembling ovarian sex cord tumors (UTROSCT) are uterine neoplasms of unknown histiogenesis which show near complete differentiation towards ovarian sex cord elements and are postulated to arise from pluripotential uterine mesenchymal cells or endometrial stromal cells with secondary sex cord differentiation.

**Case presentation:**

A 48 year old post-menopausal women presented with abnormal uterine bleeding for which she underwent total abdominal hysterectomy with bilateral salpingo-ophorectomy. Gross examination revealed a gelatinous grayish white tumor confined to the myometrium, 7 cm in maximum dimention. Microscopic examination revealed monomorphic round to oval tumor cells in anastomosing cords and trabeculae with myxoid background. Immunohistochemically, tumor cells showed diffuse positivity for vimentin, CD99 and S100, while focal positivity was seen with pancytokeratin immunostain. The case was diagnosed as UTROSCT. No evidence of metastasis was found on systemic clinical and radiologic workup.

**Conclusion:**

UTROSCT are rare uterine tumors which can be diagnosed with certainty on morphologic and immunohistochemical grounds. It is important to recognize these tumors as they behave differently from endometrial stromal tumors with sex cord like elements (ESTSCLE).

## Introduction

Heterologous mesodermal tumors of the uterus first came to clinical attention when Morehead and Bowman described a case of a uterine neoplasm resembling granulosa cell tumor in 1945 [1]. Later on in 1976, Clement and Scully further clarified the concept of sex cord differentiation of uterine tumors and categorizes them into two distinct subgroups. The first group termed as endometrial stromal tumor with sex cord like elements (ESTSCLE) largely resembles traditional endometrial stromal tumors with focal sex cord differentiation. The second group comprises of tumors entirely composed of elements resembling sex cord tumors of ovary and is named as uterine tumors resembling ovarian sex cord tumor (UTROSCT) [2]. Although seems similar these two groups of tumors differ each other significantly in terms of clinical behavior and molecular genetic features. The later group lacking typical JAZF1-JJAZ1 translocation seen in endometrial stromal tumors [3]. We describe a rare case of UTROSCT with its clinicopathologic and immunohistochemical features.

## Case presentation

**Figure 1:**
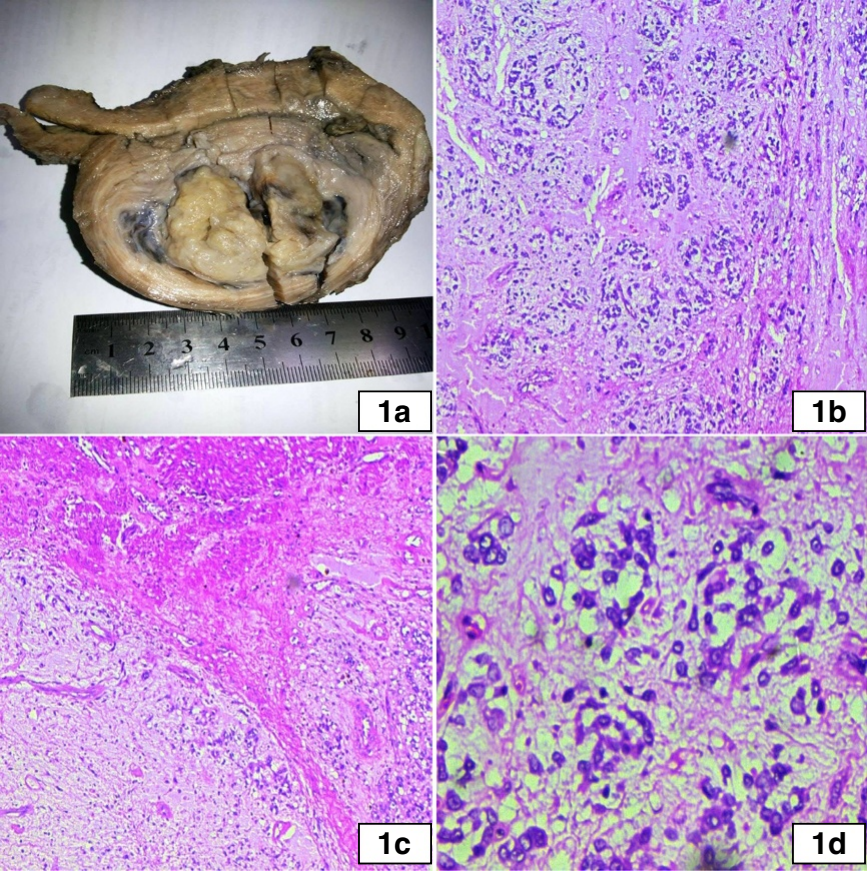
**Gross and microscopic view of UTROSCT. a**: Gross view of hysterectomy specimen showing a gelatinous grayish white tumor confined to the myometrium. **b**: Microscopic section of the tumor showing anastomosing cords and trabeculae with myxoid background. **c**: Tumor interface with myometrium revealing invasive borders of the tumor. **d**: High power microscopic section of the tumor showing round to oval cells with vesicular nuclei and pale cytoplasm.

48 year old post-menopausal women presented with abnormal uterine bleeding for more than 6 months unresponsive to hormonal therapy. After preoperative workup, she underwent total abdominal hysterectomy and bilateral salpingo-ophorectomy. The specimen was sent to histopathology laboratory. On gross examination, 7 × 5 × 4 cm mass was seen in myometrium. Overlying endometrium was grossly unremarkable and tumor was 2 mm was from serosal surface of the uterus. The tumor was completely confined to the myometrium. Cut surface of the tumor was gelatinous and grayish white (Figure 1a). Both ovaries were normal in size on gross examination. Microscopic sections of the tumor revealed anastomosing trabecule and cords of cells with retiform architecture at places (Figure 1b). Tumor showed focally invasive borders (Figure 1c). Background was myxoidy and areas of infarction were noted. Tumor cells showed vesicular nuclei with inconspicuous nucleoli and moderate amount of pale cytoplasm (Figure 1d). Overlying endometrium showed atrophic pattern with inactive glands and compact stroma. Both ovaries were unremarkable on histologic examination.

**Figure 2:**
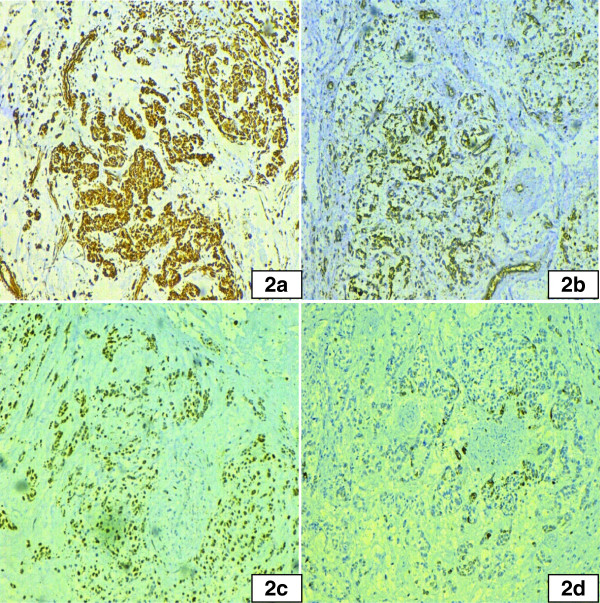
**Immunohistochemical stains of UTROSCT. a**: Vimentin showing diffuse strong positivity in tumor cells. **b**: CD99 revealing diffuse positivity in tumor cells. **c**: S100 immunostain with diffuse expression in tumor cells. **d**: Cytokeratin stain showing focal expression in tumor cells.

Immunohistochemical stains were performed by DAKO envision method on a representation section of the tumor. Tumor cells showed diffuse positivity for vimentin (Figure 2a), CD99 (Figure 2b) and S100 (Figure 2c) stains. Focal positivity was noted with pancytokeratin immunostain (Figure 2d) and occasional cells showed desmin positivity. Negative stains include ASMA, CD10, EMA, CK7, CD31, Chromogranin A, HMB45, Melan A, Calretinin, PLAP and CD117 stains. Inhibin was also done which was found to be negative. The case was also consulted with extra-institutional senior pathologists and diagnosed as UTROSCT. There was no evidence of metastasis on systemic radiologic workup.

## Discussion

Rarity of UTROSCT is reflected by the fact that no more than 70 cases have been reported to date, although more than half a decade has passed since its identification. This is the reason why its origin and pathogenesis is still a question of debate. An origin from endometrial stromal cells with sex cord differentiation has been postulated. In an ultrastructural study involving 13 cases, features of smooth muscle differentiation were lacking and therefore it was proposed that UTROSCT are polyphenotypic neoplasms with variable sex cord and focal epithelial differentiation. They either arise from endometrial stromal tumors as a result of divergent differentiation or may represent a distinct group of uterine tumors with histiogenesis related to ovarian sex cord tumors [4]. Czernobilsky suggested an origin of UTROSCT from pluripotential uterine mesenchymal cells [5].

Clinical behavior of UTROSCT differs widely from their closely related ESTSCLE. The later behaves similar to endometrial stromal sarcoma with a propensity for wide local and distant metastasis. On the other hand UTROSCT are benign tumors with occasional occurrence of local recurrence. Recently, Umeda et al. described 2 cases of UTROSCT with metastasis to pelvic lymph node and appendix respectively [6]. Mačák et al also described a similar case with lymph node metastasis; however this is largely an unusual occurrence in these tumors [7]. In our case no regional or distant metastasis was observed. Apart from clinical outcome, ESTSCLE like endometrial stromal tumors show JAZF1-JJAZ1 translocation which are absent in UTROSCT. These observations suggest a different pathogenesis of UTROSCT.

Morphologically UTROSCT are entirely composed of elements simulating ovarian sex cord tumors. They may show a variety of architectural patterns like anastomosing trabeculae, plexiform cords, tubules, microfollicles, glomeruloid structures and retiform isands [8, 9]. This may lead to difficulty in differentiating from its mimickers which include epitheloid and plexiform leiomyoma, plexiform tumorlet and metastatic ovarian sex cord stromal tumors. Moreover, endometrial stromal sarcoma and endometroid carcinoma may also show focal sex cord elements, therefore thorough sampling remains cornerstone to reach a correct diagnosis.

Epitheloid leiomyoma is composed of round to polygonal epitheloid cells and can sometimes be confused with UTROSCT morphologically, however the typical plexiform and trabecular pattern characteristic of UTROSCT is absent and the distinction can be made easily on immunohistochemical grounds. Epitheloid leiomyoma will show diffuse positivity for ASMA and lack expression of calretinin, inhibin, CD99 and melan A. Vascular plexiform leiomyoma is another variant of leiomyoma, which owing to its anastomosing trabecular architecture comes under the differential diagnosis of UTROSCT. However, positivity for smooth muscle markers and absence of expression of sex cord markers will lead to a correct diagnosis. From a prognostic standpoint, it’s more important to differentiate UTROSCT from low grade endometrial stromal sarcoma and endometroid carcinoma with sex cord differentiation. Endometrial stromal sarcoma has a typical infiltrative pattern of growth and show diffuse expression with CD 10 immunostain. Endometroid carcinoma may show sex cord differentiation but usually areas with morphology typical of endometroid carcinoma are present. Moreover, endometroid carcinoma will show diffuse positivity with pancytokeratin and EMA and negativity with WT1. Additionally, metastatic ovarian sex cord tumors should always be ruled out before rendering a diagnosis of primary UTROSCT, on clinical and morphologic grounds. Lastly, some soft tissue sarcomas like extraskeletal myxoid chondrosarcoma and synovial sarcoma may arise in the uterine wall as primary tumors. Extraskeletal myxoid chondrosarcoma can histologically resemble UTROSCT, however will not show expression of sex cord markers. On the other hand, synovial sarcoma usually show spindle cell morphology but can show areas of epitheloid differentiation. Immunohistochemical expression of EMA, CK7, Bcl2 will help in differentiation from UTROSCT along with negative expression of sex cord markers. It is important to diagnose UTROSCT accurately as most of the differentials including endometrial stromal sarcoma, endometroid carcinoma and soft tissue sarcomas are malignant and need aggressive treatment, in contrast to UTROSCT which typically behaves in an indolent fashion.

Apart from markers of sex cord differentiation, a variety of epithelial, smooth muscle and stromal markers have been reported in these tumors [10, 11]. De leval et al described immunohistochemical profile of 12 cases of UTROSCT. Half of these tumors expressed one or more sex cord markers, epithelial markers and CD10 while 11 out of 12 expressed smooth muscle markers. Among sex cord markers, inhibin was expressed in 3 of 12, calretinin in 4 of 12, WT1 in 4 of 12, and melan-A in 3 of 11 cases. CD117 and S100 were expressed in 4 and 2 cases respectively [12]. Irving et al similarlarly described immunohistochemical profile of five cases of UTROSCT. In their series, calretinin was expressed in all 5, CD99 in 4 while melan A and inhibin in 2 cases respectively [13]. Krishnamurthy et al elaborated 7 cases of UTROSCT, all of 7 expressing CD99 while melan A and inhibin were positive in 4 and 3 cases respectively [14]. Oliva et al described CD99 expression in 4 out of 7 cases and inhibin in 1 out of 7 cases [15]. Our case also showed diffuse expression of CD99, therefore it may be a sensitive marker for UTROSCT. Our case also showed diffuse expression of S100 which was not widely sought previously.

## Conclusion

In conclusion, UTROSCT are uterine tumors of unknown histiogenesis. They display a variety of architectural patterns and expresses wide range of expression for epithelial, stromal and sex cord markers. Their recognition is necessary since these tumors usually behave in a benign fashion and therefore hysterectomy is curative.

### Consent

Written informed consent was obtained from the patient for publication of this Case report and accompanying images. A copy of the written consent is available for review by the Editor-in-Chief of this journal. Ethics committee of Liaquat National hospital approved the study.
